# Traumatic intraperitoneal rupture of a hepatic hydatid cyst presenting as refractory anaphylactic shock: a rare case report

**DOI:** 10.1093/jscr/rjag383

**Published:** 2026-05-24

**Authors:** Rafil A Bshara, Rani A Bshara, Salih Hussein Ibrahim, Mustafa Shukri Almufti

**Affiliations:** Azadi Teaching Hospital, Emergency Medicine and Trauma, Duhok 42001, Iraq; Azadi Teaching Hospital, Emergency Department, Duhok 42001, Iraq; Azadi Teaching Hospital, General Surgery, Duhok 42001, Iraq; Azadi Teaching Hospital, General Surgery, Duhok 42001, Iraq

**Keywords:** hydatid cyst, *Echinococcus granulosus*, traumatic rupture, anaphylactic shock, eFAST and case report

## Abstract

Traumatic rupture of a hepatic hydatid cyst is an exceptionally rare and life-threatening surgical emergency, particularly in endemic regions as Kurdistan region, Iraq. We report the case of a 19-year-old male who presented with acute right upper quadrant pain following an initially undisclosed abdominal assault. Despite unremarkable initial radiographs and laboratory investigations, bedside point-of-care ultrasound and subsequent computed tomography revealed a ruptured hepatic hydatid cyst with the characteristic ‘water lily sign’. The patient rapidly developed severe anaphylactic shock, requiring aggressive resuscitation with adrenaline and corticosteroids. Emergency laparotomy confirmed a ruptured hepatic hydatid cyst, which was managed with germinal layer removal, deroofing, and peritoneal irrigation. The patient made an uneventful recovery and remains disease-free at 30-month follow-up. This case underscores that anaphylaxis may be the primary manifestation of occult hydatid disease following trauma, necessitating a high index of clinical suspicion even in the absence of a known history.

## Introduction

Cystic echinococcosis (ce), caused by the larval stage of *Echinococcus granulosus*, remains a significant public health concern in endemic regions, including Iraq and the Kurdistan region, where surgical incidences as high as 6.3 per 100 000 inhabitants have been reported [1]. The parasite’s life cycle typically involves canines as definitive hosts and sheep or cattle as intermediate hosts. Humans act as accidental intermediate hosts through ingestion of soil, water, or food contaminated with echinococcal eggs. The liver is the most frequently involved organ, accounting for ~70% of cases [2]. While many hepatic hydatid cysts remain asymptomatic for years, they carry a risk of serious complications, including secondary infection, intrabiliary rupture, and intraperitoneal rupture [3].

Intraperitoneal rupture is a rare but critical complication, occurring in ~1%–8% of cases [4]. Such ruptures can be spontaneous or more rarely triggered by blunt abdominal trauma. The sudden release of highly antigenic hydatid fluid into the peritoneal cavity can precipitate hypersensitivity reactions, ranging from mild urticaria to fatal anaphylactic shock [5]. In trauma cases, diagnosis is often challenging as clinical presentation may be masked by other injuries or the patient may be unaware of the underlying cystic disease. We present an exceptionally rare case where anaphylactic shock was the sentinel sign of a ruptured hepatic hydatid cyst following minimal trauma in a previously undiagnosed patient.

## Case report

A 19-year-old male with an unremarkable medical history presented to the emergency department with sudden-onset, severe right upper quadrant (RUQ) pain. He initially denied any history of trauma or preceding triggers and reported no nausea or vomiting. On admission, vital signs were stable: blood pressure 125/75 mmHg, pulse 90 bpm, and oxygen saturation 97% on room air. Abdominal examination revealed a soft, non-distended abdomen with tenderness localized to the RUQ. No guarding or rebound tenderness was present.

**Figure 1:**
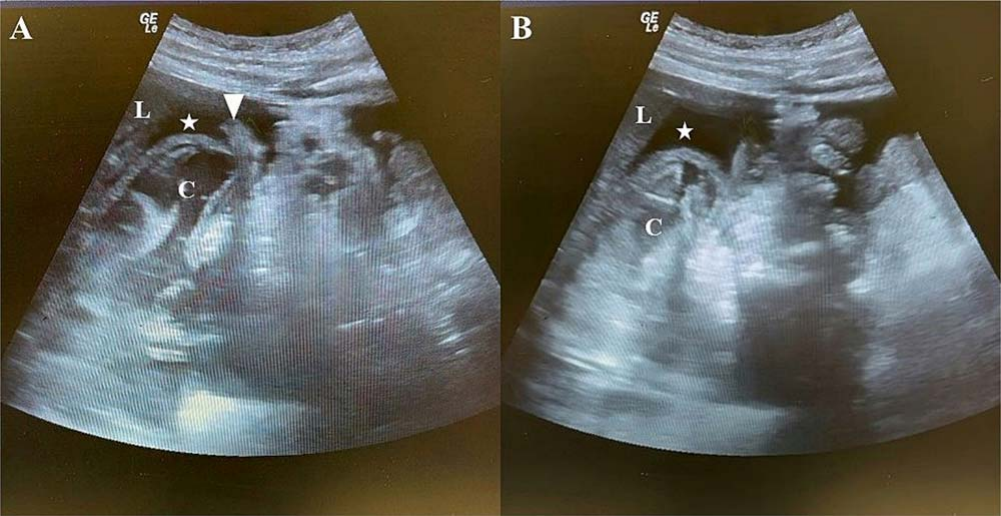
Bedside eFAST of the right upper quadrant. (A, B) eFAST images demonstrating a large anechoic fluid collection in the hepatorenal recess (Morrison’s pouch) and a complex cystic lesion is seen beneath the liver (suspected hepatic origin), containing an undulating membrane. (A) Additionally, loops of small bowel are visible floating within the fluid (arrowhead). L = liver; C = cystic lesion; asterisk (*) = free fluid collection; arrowhead = small bowel loops.

Initial laboratory investigations, including complete blood count, liver and renal function tests, lipase, electrolytes, and urinalysis, were within normal limits. Upright chest and supine abdominal radiographs were normal. Despite multimodal analgesia, the patient’s pain intensified. Given the refractory pain and unremarkable initial workup, a bedside extended Focused Assessment with Sonography for Trauma (eFAST) was performed, revealing a complex cystic lesion with an internal undulating membrane at right upper quadrant, possibly relating to the liver, with massive intraperitoneal free fluid containing freely floating small bowel loops (Fig. 1). A large anechoic fluid collection was observed at the pelvis too (Supplementary Video 1).

**Figure 2:**
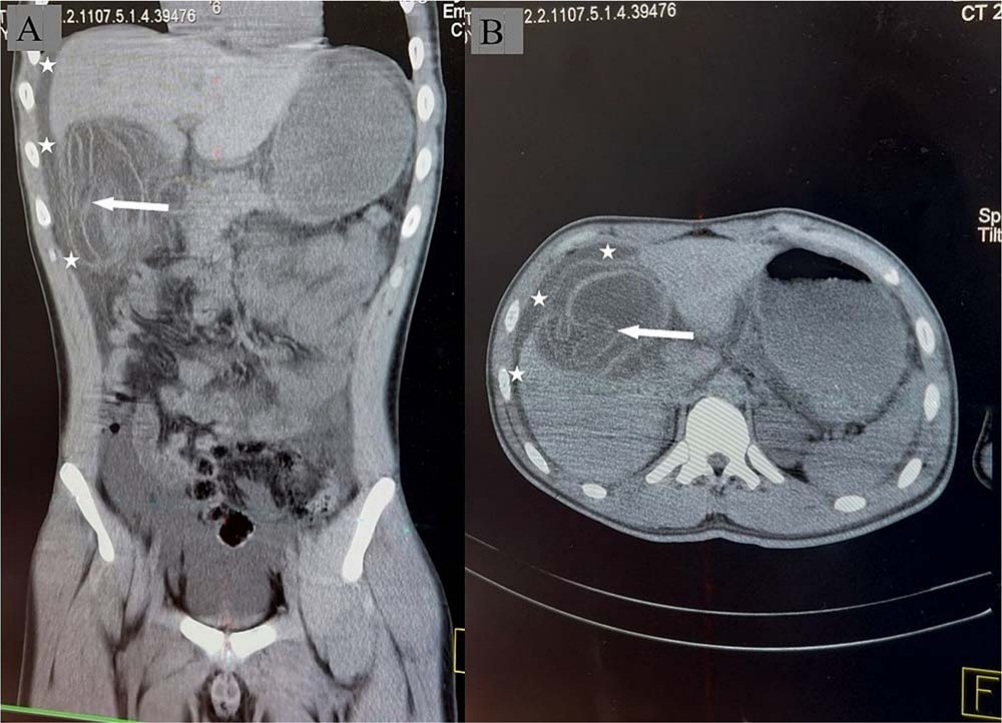
Non-contrast CT of the abdomen demonstrating a ruptured hepatic hydatid cyst. (A) coronal view showing the craniocaudal extent of the complex cystic lesion with undulating membrane (arrow). Free fluid is seen tracking from the subhepatic region to the subphrenic space (asterisk). (B) Axial view demonstrating the cystic lesion in the liver with the undulating laminated membrane the ‘water-lily sign’ being visible within the cyst cavity (arrow). Free fluid is present at the perihepatic regions (asterisk). No intervening liver parenchyma is seen between the undulating membrane of the cystic lesion and the peritoneal cavity, highly suggesting rupture to the peritoneum.

An urgent abdominal computed tomography (CT) scan strongly supported the diagnosis, demonstrating a large, irregular cystic lesion in hepatic segment V. The scan suggested a ruptured hepatic hydatid cyst, showing a detached, undulating endocyst membrane characteristic of the pathognomonic ‘water lily sign’ with extensive free fluid collections in the perihepatic spaces, including subhepatic, subphrenic, and hepatorenal recesses, confirming massive intraperitoneal rupture (Fig. 2). Shortly after returning from CT, the patient developed generalized erythema, pruritus, profound hypotension, and tachycardia consistent with anaphylactic shock. His chest remained clear on auscultation. At this point, the patient admitted recent abdominal trauma. He was managed promptly with high-flow oxygen and five sequential intramuscular doses of adrenaline (0.5 mg each of 1:1000 dilution) alongside aggressive intravenous fluid resuscitation. Adjunctive intravenous hydrocortisone and diphenhydramine were administered.

Once hemodynamically stabilized, the patient underwent emergency midline laparotomy. Over a liter of serosanguinous fluid was encountered upon entering the peritoneal cavity. Exploration revealed a ruptured hydatid cyst involving liver segments IV and V. No active bile leak was identified. The laminated germinal layer was carefully removed, and pericyst deroofing was performed. The peritoneal cavity was irrigated with aqueous 0.04% chlorhexidine gluconate, allowed to dwell for 10 minutes for maximum scolicidal efficacy, followed by thorough washing with 4 liters of warm normal saline. Two closed-suction drains were placed: one in the subhepatic space and one in the pelvis.

Postoperatively, the patient was monitored in the Intensive Care Unit. He received broad-spectrum intravenous antibiotics (ceftriaxone and metronidazole) and continued corticosteroids for 48 hours. Oral albendazole (400 mg twice daily) was initiated on postoperative day one [6]. The subhepatic and pelvic drains remained clear with minimal output and no biliary leakage, removed prior to discharge. The patient made an uneventful recovery and was discharged on day 7 postoperatively on a 6-month course of albendazole. At 30-month follow-up, he remains asymptomatic with no clinical or radiological evidence of disease recurrence.

## Discussion

Anaphylaxis following blunt abdominal trauma is an exceptionally rare clinical entity. In this instance, it served as the first, only, and most severe sign of an underlying hepatic hydatid cyst [7]. Hydatid disease frequently remains clinically silent until an acute complication, such as rupture, occurs. When complicated by profound anaphylaxis, it poses a life-threatening emergency demanding medical resuscitation and definitive surgical control.

This case highlights a significant diagnostic challenge: traumatic rupture is an uncommon presentation of echinococcosis, becoming remarkably difficult to diagnose when no obvious etiology is present or when the history of trauma is concealed by the patient [8]. In the absence of reported injury, the initial presentation of severe, isolated RUQ pain was highly deceptive. Successful management pivoted on bedside ultrasonography. The eFAST examination rapidly shifted the clinical trajectory by identifying the complex cystic lesion with large collection, allowing immediate correlation of the CT ‘water lily sign’ with the patient’s sudden hemodynamic collapse [9].

Surgically, the priority in ruptured cases is evacuation of antigenic cystic contents and prevention of secondary hydatidosis [10]. The use of a scolicidal agent like chlorhexidine gluconate, paired with a 10-minute dwell time and large-volume lavage, proved effective and safe in the absence of biliary communication [11]. Integration of prompt adrenaline-based resuscitation, meticulous surgical deroofing [12], and prolonged postoperative albendazole therapy resulted in excellent long-term outcome. This case underscores the necessity for emergency and surgical clinicians to maintain high suspicion for ruptured hydatid disease in patients presenting with unexplained shock or sudden abdominal pain, particularly in endemic regions.

## Supplementary Material

rjag383_Supplemental_Files
