# Supplementary material for: Traumatic intraperitoneal rupture of a hepatic hydatid cyst presenting as refractory anaphylactic shock: a rare case report
Source: J Surg Case Rep. 2026 May 24;2026(5):rjag383. doi: 10.1093/jscr/rjag383 (PMC13198715; doi:10.1093/jscr/rjag383)
Supplement: rjag383_Supplemental_Files [file rjag383_supplemental_files.zip › Supplementary_Video_1_rjag383.docx]

**Supplementary Video 1**: Pelvic eFAST demonstrating intraperitoneal fluid and floating small bowel.
**Legend**: Transverse pelvic view obtained during bedside eFAST examination. The urinary bladder is visualized centrally. A large anechoic fluid collection is present within the pelvis, and loops of small bowel are seen floating freely within the fluid. These findings confirm the presence of a significant intraperitoneal fluid collection.
**Alt Text**: Ultrasound video in transverse orientation showing the urinary bladder in the center. Dark, anechoic fluid surrounds the bladder, and tubular structures representing loops of small bowel move freely within the fluid.
